# MEDICAID HOME- AND COMMUNITY-BASED SERVICES IN THE WAKE OF THE COVID-19 PANDEMIC

**DOI:** 10.1093/geroni/igae098.2799

**Published:** 2024-12-31

**Authors:** Edward Miller, Lisa Beauregard

**Affiliations:** University of Massachusetts Boston, Boston, Massachusetts, United States; Mystic Valley Elder Services, Malden, Massachusetts, United States

## Abstract

Medicaid, the joint federal-state health insurance program for people with low income, provides coverage for home and community-based services (HCBS). States have significant discretion to offer HCBS programs within federal regulations. The federal government matches state Medicaid program spending according to state per capita income, with the matching rate varying from 50% to 77.27%. The need to bolster Medicaid HCBS became evident during the COVID-19 pandemic. This recognition stemmed from the challenges of keeping people safe in nursing homes and the acute workforce shortages in the HCBS sector. This presentation examines two major federal developments and state responses in HCBS options as a result of the pandemic. The first initiative entails a one-year increase of the federal Medicaid matching rate for HCBS included in the American Rescue Plan Act championed by the Biden administration. The second initiative encompasses administrative flexibilities that permitted states to temporarily expand and modify their existing Medicaid HCBS programs. It concludes that the effects of the pandemic flexibilities and enhanced federal funding on most state HCBS programs will be limited without continued investment and leadership on the part of the federal government, a Biden administration priority. States that make the American Rescue Act and COVID-19 flexibilities initiatives permanent are states that have the fiscal resources and political commitment to expanding HCBS benefits that other states lack. States’ different approaches to bolstering Medicaid HCBS during the pandemic may contribute to widening disparities in access and quality of HCBS across states and populations who depend on Medicaid HCBS.

